# Effects of Shen-Yuan-Dan on Periprocedural Myocardial Injury and the Number of Peripheral Blood Endothelial Progenitor Cells in Patients with Unstable Angina Pectoris Undergoing Elective Percutaneous Coronary Intervention

**DOI:** 10.1155/2022/9055585

**Published:** 2022-01-07

**Authors:** Zhenmin Zhang, Wenlong Xing, Hongxu Liu, Qi Zhou, Xinyi Liu, Juju Shang

**Affiliations:** ^1^Beijing Hospital of Traditional Chinese Medicine, Capital Medical University, Beijing 100010, China; ^2^Capital Medical University, Beijing 100069, China

## Abstract

**Objectives:**

We aimed to investigate the effects of Shen-Yuan-Dan (SYD), a Chinese medicine preparation, on periprocedural myocardial injury (PMI) and the number of peripheral blood endothelial progenitor cells (EPCs) in patients with unstable angina pectoris (UA) who underwent elective percutaneous coronary intervention (PCI).

**Methods:**

Patients were randomly divided into the experimental (group A) and control (group B) groups through the random number table method. In group A, patients concurrently received the conventional western treatment and SYD orally (4 capsules/time, 3 times/d, from 3 d before surgery to 7 d after surgery). In group B, patients received conventional Western medicine treatment. Both groups underwent coronary angiography, and patients undergoing PCI were eventually included in the study. The following patient data were collected: incidence of PMI, serum CK-MB content before PCI, 4 h, 24 h, and 7 d after PCI, number of CD45dim/-CD34+CD309+ peripheral venous EPCs, and number of CD184 coexpressed EPCs. The incidence of adverse reactions and 30-day major adverse cardiovascular events (MACEs) were also recorded.

**Results:**

Sixty-two patients were finally included in this study, with 32 and 30 in groups A and B, respectively. In group A, the number of peripheral blood EPCs and the number of CD184 coexpressed EPCs at 1 h before surgery were higher than those at 3 d before surgery (37.24 ± 25.20 vs. 22.78 ± 9.60/ml; *P* < 0.001 and 23.38 ± 15.30 vs. 13.54 ± 8.08/ml; *P* < 0.001, resp.). The number of peripheral blood EPCs and number of CD184 coexpressed EPCs at 4 h after surgery were lower than those at 1 h before surgery (25.30 ± 11.90 vs. 37.24 ± 25.20/ml; *P*=0.019 and 15.38 ± 8.78 vs. 23.38 ± 15.30/ml; *P*=0.013, resp.), but there was no difference at 24 h and at 7 d after surgery in comparison with that at 1 h before surgery (*P* > 0.05). In group B, compared with that at 1 h before surgery, there existed a decline in the number of EPCs in peripheral blood and the number of CD184 coexpressed EPCs at 4 h after surgery, but without a statistical difference (*P* > 0.05). Comparing both groups, it was found that the incidence of PMI in group A was lower (6.25% vs. 26.67%; *P*=0.04), and the serum CK-MB content at 4 and 24 h after surgery was also lower than that in group B (17.33 ± 5.83 vs. 20.38 ± 4.32 U/l; *P*=0.048 and 15.79 ± 5.32 vs. 19.10 ± 4.93 U/l; *P*=0.030, resp.). The number of EPCs in peripheral blood and the number of CD184 coexpressed EPCs in group A were higher than those in group B at 1 h before surgery (37.24 ± 25.20 vs. 22.36 ± 12.26/ml; *P*=0.034 and 23.38 ± 15.30 vs. 13.12 ± 14.62/ml; *P*=0.013, resp.). In addition, there were no obvious adverse reactions and no 30-day MACEs in both groups during the trial.

**Conclusion:**

SYD can reduce PMI and promote the mobilization of EPCs in the perioperative period of elective PCI in patients with UA.

## 1. Introduction

Unstable angina (UA) is a serious coronary artery disease (CAD). Percutaneous coronary intervention (PCI) is the major revascularization strategy for patients with CAD, and approximately 5 million PCI surgeries are performed every year worldwide [1]. However, periprocedural myocardial injury (PMI) is a common complication of PCI treatment. According to the *Fourth Universal Definition of Myocardial Infarction* (2018), jointly released by the European Society of Cardiology (ESC), American College of Cardiology Foundation (ACCF), American Heart Association (AHA), and World Heart Federation (WHF), PMI is defined as <5 times 99th percentile URL increase of cTn values in patients with normal baseline values (<99th percentile URL) within 48 h after PCI, or >20% increase in cTn values in patients with a higher cTn value within 48 h after PCI [2]. Reports have shown that the incidence of PMI ranges from 14% to 52% (judged by cTnI) [3], and its severity is associated with an increased risk of major adverse cardiovascular events (MACEs), such as death, reinfarction, and revascularization [4, 5]. The study related to ARMYDA showed that preoperative loading of atorvastatin calcium could reduce the occurrence of PMI [6–8], but this result has not been effectively confirmed in the Asian population [9]. Therefore, investigating drugs that can effectively prevent PMI remains a hot spot in medical research [10].

In the process of PCI treatment, some procedures, such as balloon dilation or stent implantation, will inevitably cause local vascular endothelial injury, resulting in vascular endothelial dysfunction, which is one of the main reasons for the formation of PMI [3]. Endothelial progenitor cells (EPCs) play an important role in the repair of the vascular endothelium after PCI [11, 12]. Shen-Yuan-Dan (SYD) is a Chinese medicine preparation that is effective in the treatment of CAD [13, 14]. Previous studies have shown that SYD could reduce myocardial injury and oxidative stress levels during perioperative PCI in Chinese miniswines [15]. However, oxidative stress is closely related to the function of EPCs. When oxidative stress occurs, the ability to mobilize EPCs from the bone marrow to the peripheral blood is inhibited [16], and their ability to migrate and form the vascular endothelium is also reduced [17, 18]. SYD can reduce oxidative stress in the perioperative period of PCI, but its influence on EPCs is currently unknown. Therefore, in this study, the effect of SYD on the number and function of EPCs during the perioperative period of PCI was investigated with the aim of further exploring its mechanism of myocardial protection during the perioperative period of PCI.

## 2. Materials and Methods

### 2.1. General Information

This study was a prospective, randomized, controlled clinical trial. The participants were patients with unstable angina who were admitted to the Department of Cardiology, Beijing Hospital of Traditional Chinese Medicine affiliated to Capital Medical University in 2019.

### 2.2. Study Selection

#### 2.2.1. Diagnostic Criteria

UA is defined as myocardial ischemia at rest or on minimal exertion in the absence of acute cardiomyocyte injury/necrosis [19]. The clinical presentations include: prolonged (>20 min) anginal pain at rest, new onset (de novo) angina (class II or III of the Canadian Cardiovascular Society classification), recent destabilization of a previously stable angina with at least Canadian Cardiovascular Society Class III angina characteristics (crescendo angina), or post-MI angina.

#### 2.2.2. Inclusion Criteria

The patients were included in this study if they met the following criteria: (1) patients aged 18 to 85 years, (2) those meeting the diagnostic criteria for UA, (3) patients who consented to undergo elective coronary angiography, and (4) patients who voluntarily signed the informed.

#### 2.2.3. Exclusion Criteria

The exclusion criteria were as follows: (1) patients with severe heart, liver, and renal insufficiency, such as left ventricular ejection fraction (LVEF) <30%, serum alanine transaminase (ALT) or aspartate transaminase (AST) levels >2 times the upper normal limit, or serum creatinine (Scr) >3 mg/dl; (2) patients with severe hematologic disease or malignancy; (3) patients who have taken other traditional Chinese medicine preparations regularly within 1 month before this controlled clinical trial; and (4) those who are allergic to the drugs in the composition of SYD.

### 2.3. Case Rejection

Case rejection referred to cases that were not be included, due to the following criteria: (1) their coronary angiography (CAG) showed that the patient needed coronary artery bypass graft (CABG) or did not require PCI, or the patient refused to accept undergoing PCI; (2) those who failed to be treated with test drugs according to regulations, or to take other traditional Chinese medicine preparations during the trial.

### 2.4. Sample Size

This was an exploratory study, and there was no relevant previous data to refer to; therefore, 60 patients were proposed to be included. Considering the 10% shedding rate and the fact that approximately 30% of patients with UA in our hospital do not meet the PCI criteria after CAG, 100 patients were proposed to be screened.

### 2.5. Interventions

The patients were randomly divided into an experimental group (group A) and a control group (group B) through the random number table method. The control group was treated with standardized western medicine [19], including antiplatelet aggregation drugs (aspirin enteric-coated tablets and clopidogrel bisulfate tablets), lipid-lowering drugs (atorvastatin calcium tablets), antimyocardial ischemia drugs (nitrates drugs), *β*-receptor blockers, angiotensin-converting enzyme inhibitors (ACEI), or angiotensin II receptor antagonists (ARB). Patients in the experimental group received both the conventional western treatment and SYD orally at the same time (batch number: 1902001, specification: 0.4 g/tablet, and producer: Beijing Hospital of Traditional Chinese Medicine Affiliated to Capital Medical University). They took SYD from 3 d before the interventional treatment to 7 d after the surgery continuously with 4 capsules 3 times a day.

### 2.6. Observation Indicators

#### 2.6.1. The Primary Outcome Indicators

*(1) Incidence of PMI*. The levels of peripheral blood cTnI in each group were detected 1 h before surgery and 4 and 24 h after surgery by an automatic analyzer. PMI criteria referred to the *Fourth Universal Definition of Myocardial Infarction* (2018) [2]. The incidence of PMI (100%) = number of cases of myocardial injury in the perioperative period of PCI/total number of cases × 100%.

**Figure 1:**
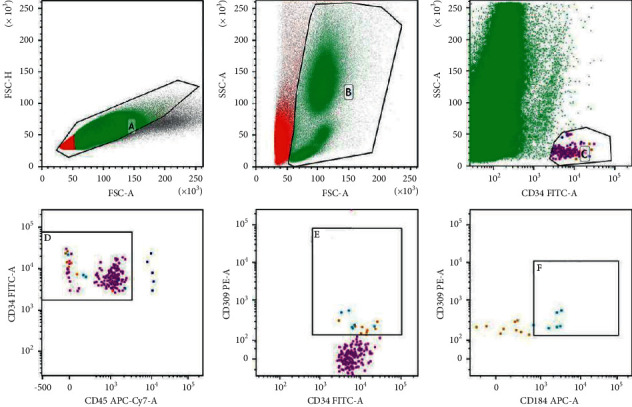
Flow diagram of EPCs flow detection. (a) Removal of adhesion cells. (b) Selection of the WBC cell population. (c) Selection of CD34+ cells. (d) Selection of CD45dim/-CD34+ cells. (e) Selection of CD45dim/-CD34+CD309+ cells. (f) Selection of CD45dim/-CD34+CD309+ CD184+ co-expressing cells.

*(2) Number of EPCs in Peripheral Blood*. The number of EPCs in peripheral blood was detected by flow cytometry. According to the 2017 EPCs naming consensus standard [20], we detected CD45dim/-CD34+CD309+ EPCs and CD184 coexpressed EPCs subgroups, respectively. We collected 2 ml of peripheral venous blood from the patients 3 d and 1 h before surgery, and 4 h, 24 h, and 7 d after surgery in an EDTA anticoagulant tube, stored at 4°C, and tested on the machine within 4 h. Three tubes were taken, and 0.5 ml peripheral blood was added into each tube. Red blood cell lysis was performed using Lysing Solution (BD Biosciences) diluted 1 : 10 (v/v) in distilled water and washed with phosphate-buffered saline (PBS) before antibody incubation. The following antibodies (BD Biosciences) were added to each test tube and incubated in the dark for 30 min at room temperature: (1) 5 *μ*l FITC anti-human-CD34, 5 *μ*l APC/Cy7 anti-human-CD45, 20 *μ*l PE Mouse IgG1, and *κ* isotype control; (2) 5 *μ*l FITC anti-human-CD34, 5 *μ*l APC/Cy7 anti-human-CD45, 20 *μ*l PE anti-human-CD309 (VEGFR2), APC Mouse IgG1, and *κ* isotype control; (3) 5 *μ*l FITC anti-human-CD34, 5 *μ*l APC/Cy7 anti-human-CD45, 20 *μ*l PE anti-human-CD309 (VEGFR2), and 5 *μ*l APC anti-human-CD184. 7-aad (BD Biosciences) was added to each test tube to remove dead cells, and the cell suspension was prepared after washing with PBS. The acquisition template was established using the BD FACS Verse software. The channel voltage was regulated by blank control, FITC Mouse IgG1 and *κ* isotype control, APC/Cy7 Mouse IgG1 and *κ* isotype control, PE Mouse IgG1 and *κ* isotype control, and APC Mouse IgG1 and *κ* isotype control-labeled specimens. BD compensation beads were used to set compensation. EPCs were obtained using the BD FACS Verse software (Figure 1).

#### 2.6.2. The Secondary Outcome Indicators

*(1) Serum CKMB Levels*. Creatine kinase-MB (CKMB) level of each patient was detected by ELISA 1 h before surgery and 4 h, 24 h, and 7 d after surgery.

*(2) Safety of the Medication and Incidence of 30-Day MACEs*. We observed the occurrence of adverse reactions, including hemorrhage (cerebral hemorrhage, gastrointestinal hemorrhage) and liver and kidney function damage within 7 d after surgery. The incidence of MACEs, including nonfatal myocardial infarction, new heart failure, and all-cause mortality, was investigated by telephone follow-up at 30 d after surgery.

### 2.7. Statistical Analysis

The verified data were analyzed using SPSS Statistical software version 27.0 (IBM Corp., Armonk, NY, USA; account name: Beijing Hospital of TCM, CCUM). The measurement data were expressed as mean ± standard deviation. The dichotomous variables were described by counting. The *t-*test was used for measurement data with normal distribution and homogeneity of variance. The approximate *t-*test was used for measurement data with normal distribution and uneven variance. Wilcoxon rank-sum test was used for measurement data with a nonnormal distribution. The Chi-squared test was used to compare the data between groups, and Fisher's exact test was used when the minimum theoretical frequency was <5. Repeated measures were compared using multivariate analysis of variance. A *P* value <0.05 was considered as statistically significant.

## 3. Results

### 3.1. Overview of Included Patients

**Figure 2:**
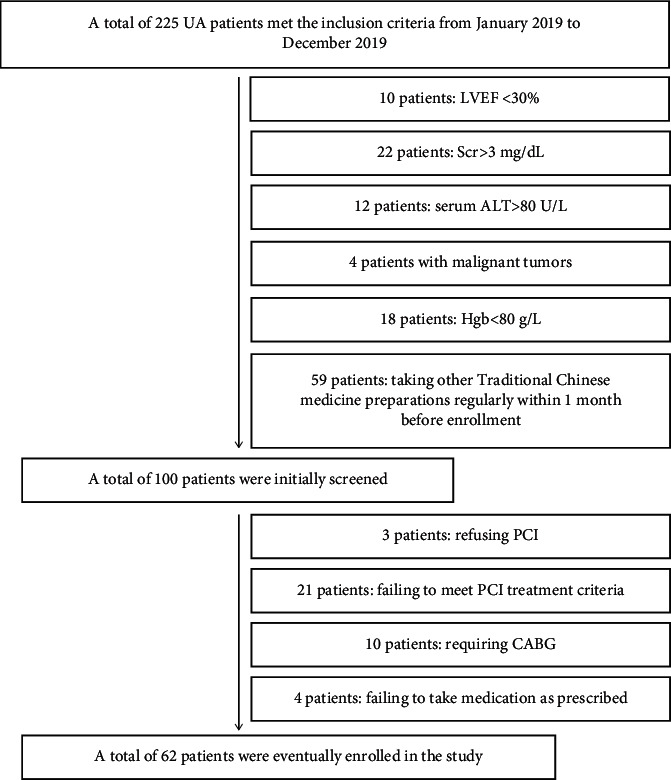
Flow diagram of patient recruitment (LVEF: left ventricular ejection fraction; Scr: serum creatinine; ALT: alanine transaminase; Hgb: hemoglobin; PCI: percutaneous coronary intervention; and CABG: coronary artery bypass grafting).

A total of 62 patients were included from January 2019 to December 2019, with 32 patients in the experimental group and 30 patients in the control group (Figure 2). There were no differences in age, sex, smoking history, clinical complications, clinical medication, blood lipid, and blood glucose between the two groups (*P* > 0.05), as shown in Table 1.

### 3.2. Effect of SYD on PMI Incidence

**Figure 3:**
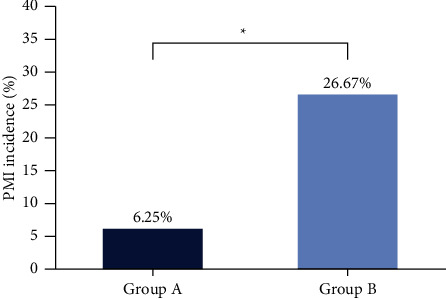
Comparison of the incidence of PMI between group A (experimental group) and group B (control group).

Among 62 participants, 6 patients had PMI. In the experimental group, the incidence of PMI was 6.25% (2/32). In the control group, it was 26.67% (8/30). The incidence of PMI in the experimental group was lower than that in the control group (*P*=0.04), as shown in Figure 3.

### 3.3. Comparison of Serum CK-MB Levels between the Two Groups during Perioperative PCI

Multivariate analysis of variance showed that there was a difference in serum CK-MB levels between the experimental and control groups (*P*=0.039). Compared with the control group, the serum CK-MB levels in the experimental group decreased at 4 and 24 h after surgery (*P*=0.048 and *P*=0.030, resp.), and there was no difference in serum CK-MB levels at 7 d after surgery (*P* > 0.05). In the experimental group, compared with the preoperative 3 d, the serum CK-MB level increased at 4 h after surgery (*P*=0.049), and there was no difference in the serum CK-MB level at 24 h and 7 d after surgery (*P* > 0.05). Compared with 4 h after surgery, serum CK-MB levels at 24 h and 7 d after surgery decreased (*P*=0.038 and *P*=0.014, resp.). In the control group, compared with 3 d before surgery, the serum CK-MB level increased at 4 h after surgery (*P*=0.008), and there was no difference at 24 h and 7 d after surgery (*P* > 0.05). Compared with 4 h after surgery, there was no difference in serum CK-MB level at 24 h after surgery (*P* > 0.05), and the serum CK-MB level at 7 d after surgery decreased (*P*=0.002), as shown in Table 2.

### 3.4. Effects of SYD on the Number of Peripheral EPCs (CD45dim/-CD34+CD309+)

**Figure 4:**
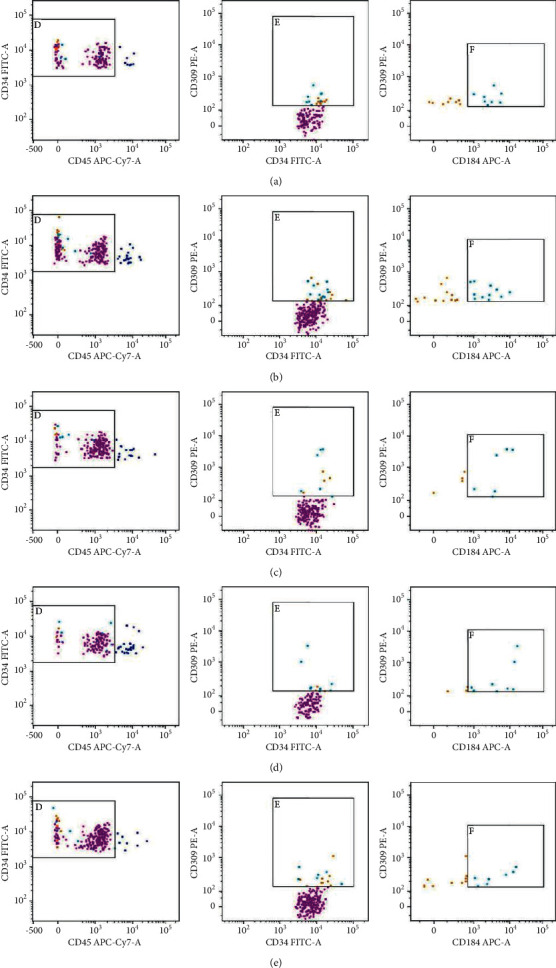
Changes in the peripheral blood EPCs number of a patient in the experimental group during perioperative PCI. (a) 3 d before surgery. (b) 1 h before surgery. (c) 4 h postoperatively. (d) 24 h postoperatively. (e) 7 d after surgery. D: CD45dim/-CD34+ cells; E: CD45dim/-CD34+CD309+ EPCs cells; and F: CD45dim/-CD34+CD309+CD184+ EPCs cells.

Multivariate ANOVA showed that the number of peripheral blood EPCs during perioperative PCI was different between the experimental and control groups (*P*=0.014). Compared with the control group, the number of peripheral blood EPCs in the experimental group increased 1 h before surgery (*P*=0.034), but no difference was found at 4 h, 24 h, and 7 d after surgery (*P* > 0.05). In the experimental group, compared with 3 d before surgery, the number of peripheral blood EPCs increased at 1 h before surgery (*P* ≤ 0.001), but there was no difference at 4 h, 24 h, and 7 d after surgery (*P* > 0.05). Compared with 1 h before surgery, the number of peripheral blood EPCs decreased at 4 h after surgery (*P*=0.019), but no difference was found at 24 h and 7 d after surgery (*P* > 0.05). In the control group, there were no differences at other time points compared with 3 d and 1 h before surgery (*P* > 0.05). More details are shown in Table 3 and Figure 4.

### 3.5. Effect of SYD on the Number of EPCs (CD45dim/-CD34+CD309+CD184+) Coexpressed by CD184 in Peripheral Blood

Compared with the control group, the number of peripheral blood CD184 coexpressed EPCs in the experimental group increased 1 h before surgery (*P*=0.013), but there was no difference in the number of peripheral blood CD184 coexpressed EPCs at 4 h, 24 h, and 7 d after surgery (*P* > 0.05). In the experimental group, compared with 3 d before surgery, the number of peripheral blood CD184 coexpressed EPCs increased at 1 h before surgery (*P* ≤ 0.001), but there was no difference at 4 h, 24 h, and 7 d after surgery (*P* > 0.05). Compared with 1 h before surgery, the number of CD184 coexpressed EPCs in peripheral blood was decreased at 4 h after surgery (*P*=0.013), but no difference was found at 24 h and 7 d after surgery (*P* > 0.05). In the control group, there were no differences at other time points compared with 3 d and 1 h before surgery (*P* > 0.05) (Table 4).

### 3.6. Safety of the Medication and Incidence of MACEs within 30 Days

During the trial, there was one patient who developed dry cough in the experimental group, while none in the control group (*P* > 0.05). There was no person suffering from bleeding and abnormal renal and liver function in the two groups within 7 d after surgery. There was no person suffering from myocardial infarction, new heart failure, or death in the two groups within 30 d.

## 4. Discussion

As a common complication of PCI treatment, the mechanism of PMI is mainly related to side branch occlusion, distal vascular embolism, vascular endothelial dysfunction, and other factors [3]. The normal vascular endothelium is composed of single-layer endothelial cells and connected with the extracellular matrix through integrins [21], which plays an important role in maintaining the balance of the coagulation and fibrinolysis system and regulating vascular tension [22]. However, PCI inevitably leads to injury or loss of endothelial cells [23–25], thereby causing local inflammatory cell infiltration [26], platelet aggregation [27], and proliferation of vascular smooth muscle cells [28], eventually leading to PMI, stent thrombosis, stent restenosis, and other complications. Due to the fact that vascular endothelial regeneration is the key to recovery of endothelial function after PCI, EPCs play an important role [29].

EPCs, a type of endothelial precursor cells derived from bone marrow, can differentiate into mature endothelial cells in vivo and in vitro [30]. They were first discovered in human peripheral blood by the Japanese scholars Asahara et al. in 1997 [31]. In the physiological state, they mainly exist in the bone marrow, and only <1% exist in the peripheral blood. They are used to supplement aging and dying endothelial cells and participate in maintaining the integrity of the body's vascular endothelial system. When vascular injury, oxidative stress, and an inflammatory reaction occur in the body, EPCs are mobilized from the bone marrow into peripheral blood and recruited to the site of ischemia or endothelial injury, participating in collateral angiogenesis and the repair of damaged vascular endothelium [32, 33]. Currently, CD45dim/-CD34+CD309+ is considered to be the main phenotype of EPCs [20, 34]. However, because CD184 plays a key role in the transendothelial migration and homing of EPCs to the site of vascular injury, EPCs with CD184 coexpression are considered to have a higher migration activity [35, 36]. Previous studies have found that the number of EPCs in peripheral blood of patients with coronary heart disease is significantly lower than that of normal people [37], while there is an increase in the incidence of adverse cardiovascular events, hospitalization rate, and mortality in patients with coronary heart disease with reduced number of EPCs [38].

The results of this study showed that the incidence of PMI in the experimental group was lower than that in the control group, and the level of CKMB in the perioperative period of PCI was also lower than that in the control group. This suggests that SYD has a myocardial protective effect in the perioperative period of PCI. To explore its mechanism of myocardial protection, we observed its effect on the number of peripheral blood EPCs during the perioperative period of PCI. The results indicated that the number of peripheral blood EPCs and the number of CD184 coexpressed EPCs in the experimental group at 1 h before surgery were higher than those at 3 d before surgery, and the number of peripheral blood EPCs and the number of CD184 coexpressed EPCs in the experimental group at 1 h before surgery were higher than those in the control group. These results suggest that SYD promotes the mobilization of EPCs in the perioperative period of PCI in patients with UA. During the trial, one patient in the experimental group developed dry cough, which was considered to be related to the use of ACEI drugs, and the symptoms disappeared after discontinuation. During the trial, no obvious bleeding, liver and kidney damage, and other adverse reactions occurred in the two groups, suggesting that SYD has superior safety in clinical application.

The change of peripheral blood EPCs number after PCI is still controversial. Previous studies have shown that the number of peripheral EPCs after PCI increased compared with that before PCI [39–41]. The results of studies by Thomas et al. [42] showed that the number of EPCs decreased by approximately 7–15% in the early postoperative period (6 h after surgery) compared with that before PCI. Montenegro et al. [43] also found that approximately two-thirds of patients had lower EPCs levels after PCI than before. The results of this study showed that the number of peripheral blood EPCs and the number of CD184 coexpressed EPCs in the experimental group decreased at 4 h after surgery compared with 1 h before surgery, which was similar to the results of Thomas and Montenegro. This may be related to the local homing of EPCs after PCI, and it has also been suggested that this may be related to the physiological changes in the number of normal peripheral blood EPCs at different time points each day [44].

However, this study still has some limitations. First, this study is an exploratory study with a small clinical sample size. Second, only cTnI and CKMB markers of myocardial injury were detected in this study, with few clinical efficacy indicators. In addition, since this study was a clinical trial, myocardial tissue could not be obtained; thus, the homing of EPCs in local tissues could not be observed, nor could the pathological changes of myocardial tissue and local blood vessels be observed. In the future, the research group needs to conduct further studies to observe the effect of SYD on EPCs homing and explore the mechanism of its effect on the number of EPCs during perioperative PCI.

In conclusion, SYD can reduce myocardial injury during the perioperative period of elective PCI in patients with UA patients and promote the mobilization of EPCs. The mechanism of its effect on the number of EPCs during perioperative period of PCI remains to be further investigated.

## Figures and Tables

**Table 1:** Comparison of the clinical characteristics between group A (experimental group) and group B (control group).

Items	Group A (*n* = 32)	Group B (*n* = 30)	*P*
Age (years)	66.59 ± 9.86	66.14 ± 10.03	0.877
Male (case)	20	17	0.640
Smoking (case)	22	22	0.691
Drinking (case)	18	16	0.818
Hypertension (case)	25	17	0.071
T2DM (case)	14	9	0.263
Hyperlipemia (case)	18	17	0.974
OMI (case)	7	9	0.465
HF (case)	6	6	0.901
ACEI/ARB (case)	30	29	0.593
Statins (case)	32	30	-
*β*-Blocker (case)	28	29	0.355
Aspirin (case)	32	30	—
Clopidogrel (case)	32	30	—
HbA1c (%)	6.67 ± 1.44	6.61 ± 1.14	0.581
CHO (mmol/L)	3.89 ± 0.84	4.14 ± 1.13	0.218
LDL-C (mmol/L)	2.32 ± 0.70	2.48 ± 0.90	0.181

ACEI: angiotensin-converting enzyme inhibitor; ARB: angiotensin II receptor blocker; HbA1c: hemoglobin A1c; CHO: cholesterol; and LDL-C: low-density lipoprotein cholesterol.

**Table 2:** Comparison of serum CK-MB levels between the two groups during perioperative PCI ($\bar{x}\pm s$, U/L).

Group	Before surgery	4 h after surgery	24 h after surgery	7 d after surgery
A (*n* = 32)	15.97 ± 4.44	17.33 ± 5.83^#*∗*^	15.79 ± 5.32^#Δ^	15.89 ± 4.77^Δ^
B (*n* = 30)	17.86 ± 3.95	20.38 ± 4.32^*∗*^	19.10 ± 4.93	17.70 ± 3.50^Δ^

A: experimental group; B: control group. ^#^*P* < 0.05 in comparison with the control group; ^*∗*^*P* < 0.05 in comparison with preoperative; and ^Δ^*P* < 0.05 in comparison with 4 h after surgery.

**Table 3:** Number of peripheral blood EPCs during perioperative PCI in the two groups ($\bar{x}\pm s$, per ml).

Group	3 d before surgery	1 h before surgery	4 h after surgery	24 h after surgery	7 d after surgery
A (*n* = 32)	22.78 ± 9.60	37.24 ± 25.20^#*∗*^	25.30 ± 11.90^Δ^	28.84 ± 19.34	26.84 ± 18.02
B (*n* = 30)	20.88 ± 7.34	22.36 ± 12.26	19.26 ± 9.82	22.00 ± 13.76	20.00 ± 8.46

A: experimental group; B: control group. ^#^*P* < 0.05 in comparison with the control group; ^*∗*^*P* < 0.05 in comparison with preoperative 3 d; and ^Δ^*P* < 0.05 in comparison with preoperative 1 h.

**Table 4:** Number of EPCs coexpressed by peripheral blood CD184 during perioperative PCI in the two groups ($\bar{x}\pm s$, per ml).

Group	3 d before surgery	1 h before surgery	4 h after surgery	24 h after surgery	7 d after surgery
A (*n* = 32)	13.54 ± 8.08	23.38 ± 15.30^#*∗*^	15.38 ± 8.78^Δ^	17.76 ± 10.48	17.22 ± 11.12
B (*n* = 30)	12.50 ± 9.26	13.12 ± 14.62	12.74 ± 9.20	12.62 ± 11.76	11.50 ± 8.78

A: experimental group; B: control group. ^#^*P* < 0.05 in comparison with the control group; ^*∗*^*P* < 0.05 in comparison with preoperative 3 d; and ^Δ^*P* < 0.05 in comparison with preoperative 1 h.

## Data Availability

The data used to support the findings of this study are available from the corresponding author upon request.

## References

[1] Faxon D. P., Leopold J. A., Abbott J. D., McElhinney D. B., Williams D. O. (2018). Circulation: cardiovascular interventions: the first 10 years. *Cardiovascular Interventions*.

[2] Thygesen K., Alpert J. S., Jaffe A. S. (2018). Fourth universal definition of myocardial infarction (2018). *Global Heart*.

[3] Bulluck H., Paradies V., Barbato E. (2021). Prognostically relevant periprocedural myocardial injury and infarction associated with percutaneous coronary interventions: a consensus document of the ESC working group on cellular biology of the heart and European association of percutaneous cardiovascular interventions (EAPCI). *European Heart Journal*.

[4] Zeitouni M., Silvain J., Guedeney P. (2018). Periprocedural myocardial infarction and injury in elective coronary stenting. *European Heart Journal*.

[5] Feldman D. N., Kim L., Rene A. G., Minutello R. M., Bergman G., Wong S. C. (2011). Prognostic value of cardiac troponin-I or troponin-T elevation following nonemergent percutaneous coronary intervention: a meta-analysis. *Catheterization and Cardiovascular Interventions*.

[6] Pasceri V., Patti G., Nusca A., Pristipino C., Richichi G., Di Sciascio G. (2004). Randomized trial of atorvastatin for reduction of myocardial damage during coronary intervention: results from the ARMYDA (atorvastatin for reduction of MYocardial damage during angioplasty) study. *Circulation*.

[7] Patti G., Pasceri V., Colonna G. (2007). Atorvastatin pretreatment improves outcomes in patients with acute coronary syndromes undergoing early percutaneous coronary intervention: results of the ARMYDA-ACS randomized trial. *Journal of the American College of Cardiology*.

[8] Ricottini E., Madonna R., Grieco D. (2016). Effect of high-dose atorvastatin reload on the release of endothelial progenitor cells in patients on long-term statin treatment who underwent percutaneous coronary intervention (from the ARMYDA-EPC study). *The American Journal of Cardiology*.

[9] Jang Y., Zhu J., Ge J., Kim Y.-J., Ji C., Lam W. (2014). Preloading with atorvastatin before percutaneous coronary intervention in statin-naïve Asian patients with non-ST elevation acute coronary syndromes: a randomized study. *Journal of Cardiology*.

[10] Li X., Lai X.-L., Fei Y.-T. (2019). Efficacy and safety of shen-yuan-dan capsules for peri-procedural myocardial injury following percutaneous coronary intervention: study protocol for a randomized, double-blind, placebo-controlled trial. *Annals of Translational Medicine*.

[11] Hu C. H., Ke X., Chen K., Yang D. Y., Du Z. M., Wu G. F. (2013). Transplantation of human umbilical cord-derived endothelial progenitor cells promotes re-endothelialization of the injured carotid artery after balloon injury in New Zealand white rabbits. *Chinese Medical Journal*.

[12] Hu Z., Wang H., Fan G. (2019). Danhong injection mobilizes endothelial progenitor cells to repair vascular endothelium injury via upregulating the expression of Akt, eNOS and MMP-9. *Phytomedicine: International Journal of Phytotherapy and Phytopharmacology*.

[13] Lai X., Zhang Y., Li M. (2020). The effect of shen-yuan-dan capsule on autophagy-related gene Atg13 promoter methylation and genomic methylation levels in atherosclerotic mice. *Acta Cardiologica Sinica*.

[14] Zhou M., Ren P., Zhang Y. (2019). Shen-yuan-dan capsule attenuates atherosclerosis and foam cell formation by enhancing autophagy and inhibiting the PI3K/Akt/mTORC1 signaling pathway. *Frontiers in Pharmacology*.

[15] Xing W. L., Tang Y., Liu H. X. (2016). Effects of invigorating qi and removing blood stasis on oxidative stress response of Chinese miniature pig PMI model. *World Chinese Medicine*.

[16] Aicher A., Heeschen C., Mildner-Rihm C. (2003). Essential role of endothelial nitric oxide synthase for mobilization of stem and progenitor cells. *Nature Medicine*.

[17] Wang F., Wang Y.-Q., Cao Q. (2013). Hydrogen peroxide induced impairment of endothelial progenitor cell viability is mediated through a FoxO3a dependant mechanism. *Microvascular Research*.

[18] Yao E.-H., Yu Y., Fukuda N. (2006). Oxidative stress on progenitor and stem cells in cardiovascular diseases. *Current Pharmaceutical Biotechnology*.

[19] Roffi M., Patrono C., Collet J.-P. (2016). 2015 ESC guidelines for the management of acute coronary syndromes in patients presenting without persistent ST-segment elevation: task force for the management of acute coronary syndromes in patients presenting without persistent ST-segment elevation of the European society of cardiology (ESC). *European Heart Journal*.

[20] Medina R. J., Barber C. L., Sabatier F. (2017). Endothelial progenitors: a consensus statement on nomenclature. *Stem Cells Translational Medicine*.

[21] Dejana E., Tournier-Lasserve E., Weinstein B. M. (2009). The control of vascular integrity by endothelial cell junctions: molecular basis and pathological implications. *Developmental Cell*.

[22] Vane J. R., Anggård E. E., Botting R. M. (1990). Regulatory functions of the vascular endothelium. *New England Journal of Medicine*.

[23] Pasternak R. C., Baughman K. L., Fallon J. T., Block P. C. (1980). Scanning electron microscopy after coronary transluminal angioplasty of normal canine coronary arteries. *The American Journal of Cardiology*.

[24] Block P. C., Myler R. K., Stertzer S., Fallon J. T. (1981). Morphology after transluminal angioplasty in human beings. *New England Journal of Medicine*.

[25] Gravanis M. B., Roubin G. S. (1989). Histopathologic phenomena at the site of percutaneous transluminal coronary angioplasty: the problem of restenosis. *Human Pathology*.

[26] Farb A., Sangiorgi G., Carter A. J. (1999). Pathology of acute and chronic coronary stenting in humans. *Circulation*.

[27] Wilentz J. R., Sanborn T. A., Haudenschild C. C., Valeri C. R., Ryan T. J., Faxon D. P. (1987). Platelet accumulation in experimental angioplasty: time course and relation to vascular injury. *Circulation*.

[28] Austin G. E., Ratliff N. B., Hollman J., Tabei S., Phillips D. F. (1985). Intimal proliferation of smooth muscle cells as an explanation for recurrent coronary artery stenosis after percutaneous transluminal coronary angioplasty. *Journal of the American College of Cardiology*.

[29] Crosby J. R., Kaminski W. E., Schatteman G. (2000). Endothelial cells of hematopoietic origin make a significant contribution to adult blood vessel formation. *Circulation Research*.

[30] Szmitko P. E., Fedak P. W. M., Weisel R. D., Stewart D. J., Kutryk M. J. B., Verma S. (2003). Endothelial progenitor cells: new hope for a broken heart. *Circulation*.

[31] Asahara T., Murohara T., Sullivan A. (1997). Isolation of putative progenitor endothelial cells for angiogenesis. *Science*.

[32] Hristov M., Erl W., Weber P. C. (2003). Endothelial progenitor cells: mobilization, differentiation, and homing. *Arteriosclerosis, Thrombosis, and Vascular Biology*.

[33] Hristov M., Zernecke A., Liehn E. A., Weber C. (2007). Regulation of endothelial progenitor cell homing after arterial injury. *Thrombosis and Haemostasis*.

[34] Fadini G. P., Losordo D., Dimmeler S. (2012). Critical reevaluation of endothelial progenitor cell phenotypes for therapeutic and diagnostic use. *Circulation Research*.

[35] Walter D. H., Haendeler J., Reinhold J. (2005). Impaired CXCR4 signaling contributes to the reduced neovascularization capacity of endothelial progenitor cells from patients with coronary artery disease. *Circulation Research*.

[36] Seeger F. H., Rasper T., Koyanagi M., Fox H., Zeiher A. M., Dimmeler S. (2009). CXCR4 expression determines functional activity of bone marrow-derived mononuclear cells for therapeutic neovascularization in acute ischemia. *Arteriosclerosis, Thrombosis, and Vascular Biology*.

[37] Schmidt-Lucke C., Rössig L., Fichtlscherer S. (2005). Reduced number of circulating endothelial progenitor cells predicts future cardiovascular events: proof of concept for the clinical importance of endogenous vascular repair. *Circulation*.

[38] Werner N., Kosiol S., Schiegl T. (2005). Circulating endothelial progenitor cells and cardiovascular outcomes. *New England Journal of Medicine*.

[39] Bonello L., Basire A., Sabatier F., Paganelli F., Dignat-George F. (2006). Endothelial injury induced by coronary angioplasty triggers mobilization of endothelial progenitor cells in patients with stable coronary artery disease. *Journal of Thrombosis and Haemostasis*.

[40] Santas-Álvarez M., Rodiño-Janeiro B. K., Paradela-Dobarro B. (2016). Endothelial progenitor cells mobilisation after percutaneous coronary intervention: a pilot study. *British Journal of Biomedical Science*.

[41] Gao M., Yao Q., Liu Y., Sun F., Ma Y., Sun G. (2015). Association between mobilization of circulating endothelial progenitor cells and time or degree of injury from angioplasty in patients with exertional angina: a prospective study. *Experimental and Therapeutic Medicine*.

[42] Thomas H. E., Avery P. J., Ahmed J. M. (2009). Local vessel injury following percutaneous coronary intervention does not promote early mobilisation of endothelial progenitor cells in the absence of myocardial necrosis. *Heart*.

[43] Montenegro F. S., Correia M., Muccillo F., Souza E Silva C. G., De Lorenzo A. (2018). Associations between endothelial progenitor cells, clinical characteristics and coronary restenosis in patients undergoing percutaneous coronary artery intervention. *BMC Research Notes*.

[44] Thomas H. E., Redgrave R., Cunnington M. S., Avery P., Keavney B. D., Arthur H. M. (2008). Circulating endothelial progenitor cells exhibit diurnal variation. *Arteriosclerosis, Thrombosis, and Vascular Biology*.

